# Resistance to wheat rusts identified in wheat/*Amblyopyrum muticum* chromosome introgressions

**DOI:** 10.1002/csc2.20120

**Published:** 2020-07-07

**Authors:** John P. Fellers, Angie Matthews, Allan K. Fritz, Matthew N. Rouse, Surbhi Grewal, Stella Hubbart‐Edwards, Ian P. King, Julie King

**Affiliations:** ^1^ USDA–ARS Hard Winter Wheat Genetics Research Unit Manhattan KS 66506 USA; ^2^ Department of Agronomy Kansas State University Manhattan KS 66506 USA; ^3^ USDA–ARS Cereal Disease Laboratory St. Paul MN 55108 USA; ^4^ Nottingham BBSRC Wheat Research Centre, Division of Plant and Crop Sciences, School of Biosciences University of Nottingham, Sutton Bonington Campus Loughborough LE12 5RD UK

## Abstract

Wheat (*Triticum aestivum* L.) rusts are a worldwide production problem. Plant breeders have used genetic resistance to combat these fungi. However, single‐gene resistance is rapidly overcome as a result of frequent occurrence of new virulent fungal strains. Thus, a supply of new resistance sources is continually needed, and new resistance sources are limited within hexaploid wheat genetic stocks. Wild relatives are able to be a resource for new resistance genes but are hindered because of chromosome incapability with domesticated wheats. Twenty‐eight double‐haploid hexaploid wheat*/Amblyopyrum muticum* (Boiss.) Eig introgression lines, with introgressions covering the majority of the T genome, were evaluated for resistance to *Puccinia triticina* Erikss., *P. graminis* Pers.:Pers. f.sp. *tritici* Erikss. & E. Henning, and *P. striiformis* Westend. f.sp. *tritici* Erikss.. At the seedling level, four lines were resistant to races of *P. triticina*, six lines were resistant to *P. graminis*, and 15 lines were resistant to *P. striiformis*. At the adult stage, 16 lines were resistant to *P. triticina*. Line 355 had resistance to all three rusts and line 161 had resistance to all tested races of *P. triticina*. Some of these lines will require further work to reduce the size of the introgressed segment; however, lines 92 and 355 have very small fragments and can be used directly as new resistance donors.

**Abbreviations**
dpi: days post inoculation
IT: infection type
KASP: Kompetitive allele‐specific polymerase chain reaction
LTN: leaf tip necrosis
SNP: single nucleotide polymorphism

## 1 INTRODUCTION

Wheat rusts are the most formidable group of pathogens influencing production. Three species, *P. triticina*, *P. graminis* f.sp. *tritici*, and *P. striiformis* f.sp. *tritici*, are the causal fungi for leaf (brown), stem (black), and stripe (yellow) rust, respectively. Each can be controlled with chemical fungicide applications. However, in many areas, environmental constraints limit production resulting in low profit margins, hindering the use of fungicides.

An alternative strategy is to develop varieties carrying genetic resistance to cereal rusts. The majority of rust resistance results from single major genes in a gene‐for‐gene interaction with the pathogen (Flor, 1955). There are also broad‐spectrum resistance genes, such as the pleiotropic gene *Lr34/Yr18/Sr57*, providing various levels of resistance to all three rusts (Lagudah et al., 2006). Single‐gene resistance is often effective during the first few years after a variety release. However, large acreages of genetically similar cultivars lead to selection of rust genome mutations that can overcome resistance, resulting in the emergence of new virulent races each year. One of the most notable examples is the *P. graminis* f. sp. *tritici* race Ug99, which appeared in Uganda in 1998 and is virulent on most commercial varieties (Pretorius, Singh, Wagoire, & Payne, 2000). *Puccinia striiformis* has historically been found in cooler climates such as the U.S. Pacific Northwest; however, new races adapted to higher temperatures began appearing in the U.S. Great Plains in 2000 (Milus, Kristensen, & Hovmøller, 2009). *Puccinia triticina* is found in all world regions of wheat production and >70 different races are found each year in North America alone (Kolmer, 2019; Kolmer, Long, & Hughes, 2005).

Rust resistance breeding is difficult because of the limited available sources of genetic variation within the gene pool of wheat. As a result, geneticists have turned to the wild relatives. The effort required to transfer interspecifc variation into wheat depends on the closeness of the wheat–wild relative relationship. For example, transfer from closely related species *Aegilops tauschii* Coss., the D genome progenitor, and *Triticum monococcum* L., one of the A genome progenitors of wheat, can be achieved through crossing elite germplasm with synthetic wheats followed by backcrossing. Transfer of wild accession genes occurs through normal chromosome pairing and recombination (Cox, 1998; Warburton et al., 2006; Zohary, Harlan, & Vardi, 1969). Linkage of target variation with undesirable traits can be resolved through further recombination with selection. The transfer of genetic variation from wild relatives carrying related but not identical genomes can be achieved using strategies like mutagenesis (Sears, 1977, 1993) or the removal of the *Ph1* locus, which restricts recombination to homologous chromosomes (Al‐Kaff et al., 2008).

*Lr9* is one of the earliest examples of resistance genes transferred from a wild relative with a nonhomoeologous genome. Using X‐rays, a wheat/*Ae. umbellulata* Zhuk. monosomic addition line was irradiated, and through chromosome breakage and repair, the chromosome segment with *Lr9* translocated into the wheat genome (Sears, 1956). Stem rust resistance gene *Sr2* was derived from emmer wheat [*Triticum turgidum* L. subsp. *dicoccon* (Schrank) Thell.] (McFadden, 1930). *SrTA10171* and *SrTA10187*, with resistance to Ug99, were transferred from *Ae. tauschii* (Olson et al., 2013). Resistance has been found in many relatives. Stem rust resistance has been found in hexaploid introgression lines with segments from rye (*Secale cereale* L.), *Leymus mollis* (Trin.) Pilg, *L. racemosus* (Lam.) Tzvelev, *and Thinopyrum* junceiforme (Á. Löve & D. Löve) Á. Löve (Rahmatov et al., 2016) and crosses of durum wheat [*Triticum turgidum* L. subsp. *Durum* (Desf.) van Slageren] with *Th. intermedium* (Host) Barkworth & D. R. Dewey, *Th. bessarabicum* (Savul. & Rayss) Á. Löve, *Th. elongatum* (Host) D. R. Dewey, *Th. ponticum* (Podp.) Barkworth & D. R. Dewey, *Anthosachne rectiseta* (Nees) Barkworth & S. W. L. Jacobs, *Ae. caudata* L., *and Ae. speltoides* Tausch (Xu, Jin, Klindworth, Wang, & Cai, 2009). Introgressions can also provide resistance to multiple pathogens. Resistance to all three rusts was obtained by the transfer of the short arm of chromosome 1R of rye (1RS) containing *Lr26*, *Sr31*, and *Yr9* (Zeller, 1973), while a Robertsonian translocation from *Th. intermedium* carried resistance genes *Sr44* and *Bdv2* to *P. graminis* and *Barley yellow dwarf virus*, respectively (Liu et al., 2013).

*Amblyopyrum muticum* (*Aegilops mutica* Boiss; 2*n* = 2*x* = 14; genome TT) is a wild relative of wheat originating from the Middle East and Armenia. *Am. muticum* possesses a *Ph1* suppressor gene that facilitates recombination between *Am. muticum* chromosomes and the homoeologous chromosomes of wheat (Dover & Riley, 1972). Relatively little research has previously been undertaken on *Am. muticum*, but merit as a genetic resource has been shown. Lines homozygous for a 5D/5T introgression provide winter hardiness (Iefimenko, Fedak, Antonyuk, & Ternovska, 2015), while a complete 7T chromosome substitution line provides resistance to powdery mildew (*Blumeria graminis* f. sp. *tritici*; Eser, 1998). With the development of new marker systems and higher throughput karyotyping, the majority of the *Am. muticum* genome has now been introgressed into hexaploid wheat (King et al., 2017, 2019). In this report, we begin the characterization of the first 28 lines containing overlapping segments of the T genome by screening for resistance to cereal rusts.

## 2 MATERIALS AND METHODS

### 2.1 Introgression lines

The development, and characterization of the double‐haploid wheat/*Am. muticum* lines are described in King et al. (2017, 2019) and in Supplemental Table S1. In summary, two *Am. muticum* accessions, JIC2130004 and JIC2130012, were obtained from the Germplasm Resource Unit (John Innes Centre, Norwich, UK) and initially crossed to either ‘Chinese Spring’ (CI6223) or ‘Pavon 76’ (PI519847), The F_1_ products were crossed to either ‘Paragon’ (Cereal Variety Handbook, 1997) or Pavon 76, then backcrossed to Paragon to the BC_3_. At the BC_3_ generation, double haploids were produced to stabilize the segments using maize pollination, embryo rescue, and chromosome doubling (King et al., 2019).

### 2.2 Seedling infection testing

Five seeds from each of the 28 doubled‐haploid introgression lines were planted in 20‐ by 20‐ by 3‐cm aluminum cake pans containing BM‐1 soil media (Berger Peat Moss, LLC). Five seeds of the hexaploid parents Paragon and Pavon 76 along with a susceptible check ‘Thatcher’ were also included and placed throughout the pan. At the two‐ to three‐leaf seedling stage, 25 mg of *P. triticina* rust spores from each of the races BBBD, TNRJ (PRTUS35), and 97AZ103 were suspended in 2 ml of Soltrol 170 parafin oil (Phillips Petroleum) and sprayed onto the seedlings with an atomizer (Tallgrass Solutions) at 40 psi. Seedlings were transferred to a Percival humidity chamber at 100% relative humidity with wall settings of 5 °C and water basin of 40 °C for 16 h. Plants were transferred back into the greenhouse. At 14 d post inoculation (dpi), infection types were rated on a scale of 0–4 (McIntosh, Wellings, & Park, 1995): 0, being no infection; a semicolon (;,fleck) being small focused infection points caused by hypersensitive reaction with no pustule formation; 1–4 scale of pustule size with 1 being very small and focused with distinct hypersensitive reaction to 4 being large pustules and no plant defense reaction.

A second set of plants was inoculated with a composite of two *P. striiformis tritici* isolates collected in Kansas in 2012 and 2014 (Robert Bowden, personal communication, 2018). Plants were inoculated as above but humidity chamber conditions were wall settings of 0 °C and water basin of 29 °C for 24 h. At 14 dpi, plants were scored using a scale of 1 (resistant) to 9 (susceptible) (McNeal, Konzak, Smith, Tate, & Russell, 1971).

A third set of seedlings were inoculated with a composite of two races of *P. graminis tritici*, RKQQC and QFCFC (obtained from Robert Bowden in 2018), as above except humidity chamber conditions were with wall settings of 6 °C and water basin of 42 °C for 16 h. At 14 dpi, infection types were scored on a scale of 0, ;, 1–4 (see description for leaf rust; McIntosh et al., 1995). Seedlings were also tested at the USDA–ARS Cereal Disease Laboratory (St. Paul, MN) BL‐3 containment facility with *P. graminis tritici* Ug99 Race TTKSK (isolate 04KEN156/04) as described in Hundie et al. (2019).

### 2.3 Adult infection testing

Two plants from each line including Paragon, Pavon 76, and the susceptible check Thatcher were grown in a 4‐L pot containing BM‐1 soil media in the greenhouse as described above. At spike emergence, plants were inoculated with a field composite of *P. triticina* collected in 2017 from Thatcher growing in Alfalfa County, OK, and transferred to a humidity chamber as described previously. At 21 dpi, flag leaves were scored for infection using percentage of leaf coverage and infection type (McIntosh et al., 1995).

### 2.4 *Lr34* and *Lr46* screening

Twenty‐one lines were tested for the presence of *Lr46* and *Lr34* using Kompetitive allele‐specific polymerase chain reaction (KASP) markers. Fifty to 100 ng of DNA was dried in a 384‐well plate and resuspended using 3 μl of KASP assay master mix containing primer, 2× KASP buffer and enzyme (Keygene), and MgCl_2_. For *Lr46*, the primer used was *Lr46‐Yr29*_JF2‐2‐KASP (Brown‐Guedira, G. and Fellers, J.P., unpublished), and for *Lr34*, two separate primers *Lr34*Exon11‐KASP (Lagudah et al., 2009) and *Lr34*JagExon22‐KASP (Yan, L., personal communication, 2016) were used to check for the *Lr34* gene and also confirm there was not a false‐positive gene. For *Lr34*exon11 and *Lr46*, the thermal cycler conditions used were as follows: 94 °C for 15 min, 97 °C 15 sec, 68 °C for 1 min at −0.8 °C per cycle for 10 cycles, 97 °C for 20 sec, 60 °C for 1 min for 30 cycles, 59 °C for 30 sec at −1.0 °C per cycle for 34 cycles, and a final cool down of 10 °C for 5 min. For *Lr34*JagExon22, the thermal cycler conditions were as follows: 94 °C for 15 min, 94 °C for 20 sec, 60 °C for 1 min for 30 cycles, and a cool down for 5 min at 10 °C. Reactions were run on a GeneAmp Systems 9700 (Applied Biosystems) and results were processed using Klustercaller (LGC Biosearch Technologies).

## 3 RESULTS

**FIGURE 1:**
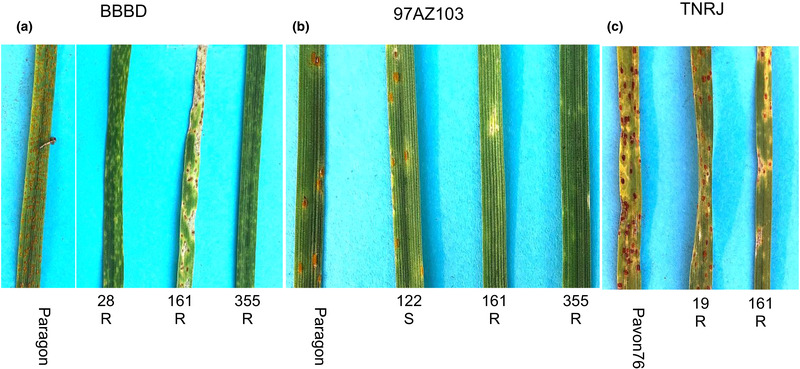
Infection types of seedling bread wheat/*Amblyopyrum muticum* introgression lines infected with *Puccinia triticina* (leaf or brown rust) races (a) BBBD, (b) 97AZ103, and (c) TNRJ. Plants were rated as resistant (R) based on infection types of 0, ;, 1, and 2 or susceptible (S) with infection type of >= 3 (McIntosh et al., 1995)

Three different races of *P. triticina* were used to screen seedlings of the introgression lines. Race 1 BBBD, the most avirulent race available with virulence only to *Lr14a*, *Lr14b*, *Lr20*, and *Lr50*, was used to identify any resistance contained in the lines. Only lines 28, 161, and 355 were resistant with infection type (IT) ; (fleck) meaning a very small, hypersensitive‐like point on the leaf with no pustule formation (Figure 1A; Table 1). Isolate 97AZ103A, originally collected from durum wheat in Arizona, is virulent on *Lr2c*, *Lr3*, *LrB*, *Lr10*, *Lr14b*, and *Lr20* and represents one of the most virulent races on U.S. durum (tetraploid) wheat. Lines 161 and 355 were resistant to 97AZ103A (Figure 1B; Table 1).

**TABLE 1:** Disease reactions of bread wheat/*Amblyopyrum muticum* introgression double‐haploid lines after infection with *Puccinia triticina* (leaf), *P. graminis tritici* (stem), and *P. striiformis tritici* (stripe). Resistance was evaluated at the seedling and adult stages. Lines were also tested for the presence of resistance gene *Lr34*

	Leaf rust						
	Seedling			Adult	Stem rust		
	BBBD	97AZ103	TNRJ	Oklahoma composite	Kansas composite	Stripe (yellow) rust	*Lr34* ^e^
Line	Infection type^a^			Infection type^b^	Infection type^c^	Infection type^d^	Exon 11
1	3+	3	3	**1–2C**	4	9	ins/ins
11	3	3	3	**1–2C**	4	9	NT
15	3	3	3	**1–2C**	4	9	ins/ins
16	3+	3	3	90S	4	2	ins/ins
17	3+	3	3	**20R, LTN**	4	5	**del/del**
19	3−	3	**2**	**0R**	4	3	ins/ins
20	3	3	3	40Z	4	9	ins/ins
21	3+	3	3	90S	**1–2**	8	ins/ins
27	3	3	3	30MR	**2**	**2**	NT
28	;	3	3	90S	4	8	NT
29	3	3	3	40MR	**; 1**	**1**	ins/ins
65	3	3	3	80S	NT	9	NT
86	3	3	3	**30 MR**	**1–2**	9	NT
92	3	3	3	**1–2C**	**2**	**1**	NT
96	3	3	3	**1**	4	9	NT
121	3	3	3	90S	4	**1**	ins/ins
122	3	3	3	**1N**	4	**4**	ins/del
123	3	3	3	90S	4	9	ins/del
124	3	3	3	90S	4	9	NT
161	**;**	**;**	**1C**	**; 0R**	4	9	ins/ins
191	3+	3	3	**1–2C**	4	**4**	ins/ins
192	3+	3	3	**5R**	4	**3**	ins/ins
195	3+	3	3	**0R**	4	**4**	ins/ins
196	3	3	3	**1C,10R**	4	**3**	ins/ins
198	3+	3	3	**5 R**	4	**3**	ins/ins
202	3	3	3	60S	4	**3**	ins/ins
348	3	3	3	90S	4	9	NT
355	**;**	**;**	3	**5R**	**1–2**	**4**	ins/ins
Pavon 76	3	3	3	90S	4	9	ins/ins
Paragon	3	3	3	1–2C	4	7	ins/ins

^a^0, ;, 1, 2, 3, where 0 is resistant (R) and 3 is susceptible (S). ^b^0R–90S, adult flag leaf based on percentage leaf coverage; LTN, leaf tip necrosis; C, chlorosis; N, necrosis; MR, medium resistance; Z, more pustules at the base, fewer at the tip (McIntosh et al., 1995). ^c^For seedlings, 0, ;, 1–4, where 0 is resistant and 4 is susceptible (McIntosh et al., 1995). ^d^1–9, where 1 is resistant and 9 is susceptible (McNeal et al., 1971). ^e^NT, not tested; ins, insertion; del, deletion.

The third isolate, TNRJ, one of the most virulent on hexaploid bread wheat, is virulent on *Lr* genes *Lr1*, *Lr2a*, *Lr2c*, *Lr3*, *Lr9*, *Lr24*, *Lr3ka*, *Lr17*, *Lr30*, *Lr10*, *Lr14a*, *Lr28*, *Lr39*, *Lr14b*, *Lr20*, *and Lr28*. Line 19 was scored as moderately resistant (IT 2) having reduced pustule sizes. Line 161 was scored resistant with very small pustules surrounded by chlorosis (IT‐1C) (Figure 1C; Table 1).

A *P. triticina* field composite, representing the current *P. triticina* population in the U.S. Great Plains, was used to screen adult plants. There were three different reaction types. First, recurrent parent Paragon exhibited an IT of 1‐2C (a reduced pustule size with a surrounding chlorotic ring) and even pustule distribution across the flag leaf. Five of the introgression lines showed this reaction (1, 11, 15, 92, and 191; Table 1). Second, four lines, 19, 161, 195, and 355, exhibited near immunity with few to zero pustules. The third set of lines, 17, 20, 27, 29, 86, and 196, had reduced total coverage of spores, without the chlorotic halos, which is more resistant than the parents. Interestingly, lines 191, 192, 195, 196, and 198 contain the same segment and all show some level of resistance, suggesting the presence of a resistance genes on 7T. Line 202, however, which also contains the same 7T segment is susceptible (see discussion). Line 17 exhibited leaf tip necrosis (LTN; Table 1) and therefore all lines were assessed for the presence of diagnostic molecular marker alleles for *Lr34* and *Lr46*, both broad‐spectrum adult plant resistance genes. Only line 17 was positive for the deletion in Exon 11 of *Lr34* (del/del; Table 1) and hence was the only line containing *Lr*34. The *Lr46* diagnostic single nucleotide polymorphism (SNP) (G) was heterozygous (G/C) in all lines except 196 and 202, which were homozygous (C/C), that is, *Lr46* absent.

**FIGURE 2:**
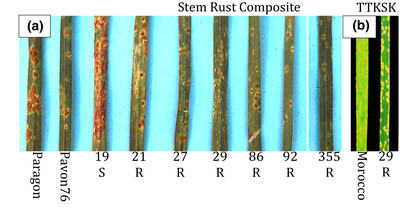
Infection types of seedling bread wheat/*Amblyopyrum muticum* introgression lines infected with *Puccinia graminis tritici* (stem or black rust). (a) Composite of QFCSC and RKQQC. (b) Ug99 race TTKSK. Plants were rated as resistant (R) based on infection types of 0, ;, 1; moderately resistant (MR) 2; moderately susceptible (MS) 3; or susceptible (S) with infection type of ≥3 (McIntosh et al., 1995)

Seedlings were tested with a composite of two *P. graminis* f. sp*. tritici* isolates commonly found in the U.S. Great Plains. RKQQC and GFCFC have a combined virulence to the following *Sr* genes: *5*, *6*, *7b*, *8a*, *9a*, *9b*, *9d*, *9g*, *10*, *17*, *21*, *36*, *Tmp*, and *McN* (Jin et al., 2008). Lines 21, 27, 86, 92 and 355 had infection types indicating differing levels of resistance, while line 29 was very resistant having an IT of ;1 (small hypersensitive regions, some with pustules; Figure 2a). Tests with TTKSK, for a possible new source of resistance to Ug99 race group (TTKSK is virulent on *Sr* resistance genes *5*, *6 7b*, *8a*, *9a*, *9b*, *9d*, *9e*, *9g*, *10*, *11*, *17*, *21*, *30*, *31*, *38*, and *McN* [Jin et al., 2008]), identified only line 29 with resistance to TTKSK (Figure 2b).

**FIGURE 3:**
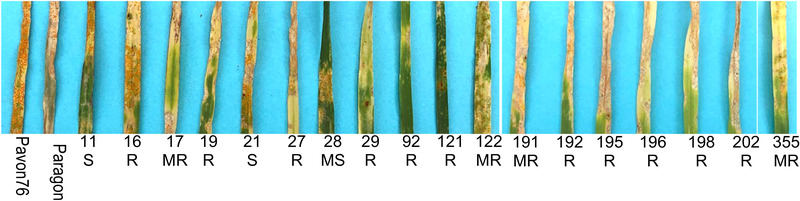
Infection types of seedling bread wheat/*Amblyopyrum muticum* introgression lines infected with a Kansas composite of *Puccinia striiformis tritici* (stripe or yellow rust). Plants were rated as resistant (R) based on infection types of 1–3; moderately resistant (MR) 4–5; moderately susceptible (MS) 6–7; or susceptible (S) with infection type of ≥8 (McNeal et al., 1971)

Resistance phenotypes to *P. striiformis tritici* have larger lesions and use a different rating scale based on lesion length and number of pustules. The composite used to inoculate the seedlings represents the current Great Plains field population and is most notable for overcoming *Yr17* and the unknown ‘TAM111’ *Yr* gene within quantitative trait loci *QYr.tamu‐2B* (Yang et al., 2019). Paragon appeared to have low level of resistance to this composite (Figure 3) and was scored as 7 out of 9 (Table 1). Fifteen of the introgression lines were scored with high to medium levels of resistance ranging from 1 to 5. Lines 16, 27, 29, 92, and 121 had the highest level of resistance (Table 1; Figure 3).

## 4 DISCUSSION

The use of wheat relatives in crop improvement has been technologically limited by an inability to quickly identify and characterize introgressions. The assumed low levels of recombination between the chromosomes of wheat and those of the wild relatives have also prevented adoption of this source of variation. However, because of the presence of a *Ph1* inhibitor in *Am. muticum* and the application of new marker technology, over 200 introgressions have been generated between wheat and *Am. muticum* (Dover & Riley, 1972; King et al., 2017, and 2019; Grewal et al., 2019). Twenty‐four of the 28 lines evaluated here carry single introgressions with the remaining four carrying either two or three. The introgressions in two of the 28 lines are whole chromosomes or very large chromosome segments from the T genome but nine are small to telomeric in size (see karyotypes in King et al., 2019). The smaller introgressions are likely to carry fewer deleterious genes and thus can be more rapidly integrated into a breeding program. Larger introgressions, however, frequently need to be reduced in size in order to minimize linkage drag. This can be done via the strategy of overlapping introgressions (Sears, 1956) or via recombination with the B and D genomes (Glémin et al., 2019). In this report, we begin assessing whether the T genome has useful resistance to the three cereal rusts.

Useful resistance was found to all three cereal rusts within the introgression lines with some of the resistance because of new resistance genes. Line 355 had seedling resistance to the less virulent *P. triticina* races and adult resistance to the field composite but was susceptible to TNRJ. Line 355 was also resistant to Great Plains isolates of *P. graminis* f. sp. *tritici* but not Ug99. Line 355 has a small 1T fragment and because of its resistances may be useful for durum and bread wheat improvement. Line 161 had very good *P. triticina* resistance at both seedling and adult stages but contains a large segment of 1T. One *Lr* gene may be shared with Line 355, but line 161 may also have a second gene that provides resistance to TNRJ. The *Am. muticum* lines also provided new resistance to *P. graminis* and *P. striiformis*. Lines 29 and 92 were highly resistant to both species, with 29 also resistant to *P. graminis* Ug99 race TTKSK. No stem rust resistance gene derived from *Am. muticum* has been previously described, therefore this resistance is new. Line 29 contains a whole 7T chromosome and thus will need to be reduced in size before it can be used in a breeding program. Unfortunately, the lines in this study with large or small recombined segments derived from 7T did not exhibit resistance to Ug99, suggesting that they are missing the fragment of the whole chromosome with the resistance gene. Line 92 contains a very small introgression from 5T (King et al., 2019).

Several of the introgression lines tested were produced from the same original BC_3_ plants (Supplemental Table S1) and were therefore expected to contain the same segments. Indeed, the molecular and cytogenetic characterization appeared to confirm this; however, some of the lines tested containing the same segments did not give the same resistance results. The most notable of these are lines 124 and 355 and line 202 compared with lines 191, 192, 195, 196, and 198. Line 124 was susceptible to all races tested while, as outlined above, line 355 shows useful resistance to leaf and stem rust. Lines 191, 192, 195, 196, and 198 all show adult resistance to *P. triticina*, while line 202 was susceptible. There are a number of possible explanations for these apparently conflicting results. Firstly, neither the molecular nor cytogenetic characterization would have revealed small differences in the size of the segments. The markers used for the characterization of the lines were part of the Axiom Wheat Wild Relative Array (King et al., 2019) and while they give good coverage of the chromosomes, gaps do exist. Secondly, additional very small segments might have been present in some lines but not detected. It has become clear that the level of recombination seen in the wheat/*Am. muticum* introgression lines is very extensive and the recombination occurs in the gametes of all generations and not just the F_1_ hybrids as originally expected.

The hexaploid heritage is also contributing some adult plant resistance in the introgression lines. Chinese Spring has previously been shown to contain *Lr34* (McIntosh et al., 2008). Line 17 exhibited LTN and was indeed positive for the active *Lr34* allele. Chinese Spring was the first cross to *Am. muticum* for several of the introgression lines and therefore likely to be the source of *Lr34* in line 17. However, it was not maintained in the other lines developed from Chinese Spring, that is, lines 15, 16, 19, 20, and 21. Pavon76, Paragon, and 24 of the introgression lines were also found to be heterozygous for the *Lr46* allele. However, the heterozygous result for the introgression lines probably is due to the functionality of the marker as these lines were produced via a doubled‐haploid procedure and were therefore expected to be homozygous at all loci. The races used for all three pathogens were all virulent on Pavon76 and Paragon with the exception of the *P. triticina* field composite, which exhibited an adult *Lr12*‐like reaction on Paragon (McIntosh et al., 1995). Paragon is listed as having moderate resistance to *P. triticina* and *P. graminis* without specific resistance gene designations (Cereals Variety Handbook, 1997). *Lr12* is known to be present in Chinese Spring, but introgression lines without Chinese Spring in their parentage also expressed this phenotype, suggesting Paragon may be the source (McIntosh et al., 2008). The infection phenotype of lines 19, 86, 161, 192, 195, 196, 198, and 355 was different than that of Paragon suggesting the presence of different genes from chromosomes 1T, 2T, and 4T or 7T.

In this work we have evaluated a group of *Am. muticum* introgression lines as a new source for resistance to cereal rusts. The screening has identified several lines with resistance to rust races that are representative of current field populations. Five lines—86, 92, 96, 121, and 355—are now a useful source of germplasm with introgressions supported by genomic in situ hybridization (GISH) (King et al., 2019) and are supported by markers to follow the introgressions. From 237 *Am. muticum*‐specific SNPs, 137 KASP markers have been derived that span the genome (Grewal et al., 2019; Supplemental Table S1 includes KASP markers that can be used for each line). Most importantly, two lines have small fragments that should have less linkage drag and can be useful as germplasm in breeding strategies. The germplasm is available through the John Innes Centre Germplasm Resource Unit or by request from USDA–ARS.

## AUTHOR CONTRIBUTIONS

J. Fellers conceived and conducted the screening experiments, provided funding, and co‐wrote the manuscript. A. Matthews and A.K. Fritz provided marker analysis and co‐wrote the manuscript. M. Rouse conducted the Ug99 screening and revised the manuscript. S. Grewal, J. King, and I.P. King provided the lines, verified the introgressions with GISH and markers and co‐wrote the manuscript.

## CONFLICT OF INTEREST

The authors declare that there are no conflicts of interest.

## Supporting information

Supplemental Table S1. Genetic background of each introgression line tested for rusts, including source of *Am. muticum* fragment, size, wheat chromosome integration site, and KASP marker to follow trait.
